# Epstein–Barr Viruses: Their Immune Evasion Strategies and Implications for Autoimmune Diseases

**DOI:** 10.3390/ijms25158160

**Published:** 2024-07-26

**Authors:** Yuehong Zhao, Qi Zhang, Botian Zhang, Yihao Dai, Yifei Gao, Chenzhong Li, Yijing Yu, Conglei Li

**Affiliations:** 1 School of Medicine, The Chinese University of Hong Kong, Shenzhen 518172, China; yuehongzhao@link.cuhk.edu.cn (Y.Z.); qizhang7@link.cuhk.edu.cn (Q.Z.); botianzhang@link.cuhk.edu.cn (B.Z.); yihaodai@link.cuhk.edu.cn (Y.D.); yifeigao2@link.cuhk.edu.cn (Y.G.); lichenzhong@cuhk.edu.cn (C.L.); 2 Ciechanover Institute of Precision and Regenerative Medicine, School of Medicine, The Chinese University of Hong Kong, Shenzhen 518172, China

**Keywords:** Epstein–Barr virus, immune evasion, autoimmune diseases

## Abstract

Epstein–Barr virus (EBV), a member of the γ-herpesvirus family, is one of the most prevalent and persistent human viruses, infecting up to 90% of the adult population globally. EBV’s life cycle includes primary infection, latency, and lytic reactivation, with the virus primarily infecting B cells and epithelial cells. This virus has evolved sophisticated strategies to evade both innate and adaptive immune responses, thereby maintaining a lifelong presence within the host. This persistence is facilitated by the expression of latent genes such as EBV nuclear antigens (EBNAs) and latent membrane proteins (LMPs), which play crucial roles in viral latency and oncogenesis. In addition to their well-known roles in several types of cancer, including nasopharyngeal carcinoma and B-cell lymphomas, recent studies have identified the pathogenic roles of EBV in autoimmune diseases such as multiple sclerosis, rheumatoid arthritis, and systemic lupus erythematosus. This review highlights the intricate interactions between EBV and the host immune system, underscoring the need for further research to develop effective therapeutic and preventive strategies against EBV-associated diseases.

## 1. Introduction

Epstein–Barr virus (EBV), the first discovered human tumor virus, is a member of the human γ-herpesvirus family [1]. It is one of the most successful viruses, infecting up to 90% of the world’s adult population and maintaining a lifelong asymptomatic infection within the B lymphocyte pool [1,2]. EBV primarily infects B cells and epithelial cells but also affects other cell types [3]. EBV primarily spreads through body fluids, such as saliva, and can also spread through blood, semen, and organ transplantations [4]. Usually, primary EBV infection in humans occurs in early childhood and is generally asymptomatic [5]. However, infection with EBV during adolescence or later can cause infectious mononucleosis [6], which can include symptoms such as fever, sore throat, and swollen lymph glands [7]. EBV facilitates the development of several kinds of cancer and autoimmune diseases as well. Numerous studies have found that EBV can be detected in patients with a variety of diseases, such as nasopharyngeal carcinomas (NPCs) [8], gastric cancer [9], Hodgkin’s disease [10], Burkitt lymphoma [11], lymphoepithelial carcinoma [12], and multiple sclerosis [13]. 

The life cycle of EBV consists mainly of a primary infection, a latent phase, and a lytic reactivation phase [14]. Upon primary infection, EBV initially targets naive B cells and drives their differentiation into memory B cells, wherein the virus establishes latency [6,11,15]. These infected memory B cells circulate throughout the body and can persist in the host for a lifetime. Moreover, EBV can switch between the latent and lytic phases [16]. Various triggers, such as stress, immunosuppression, and inflammation, can reactivate the virus and enter the lytic cycle [17,18], producing new viral particles. 

In recent years, there has been an increased focus on the mechanisms behind the long latency of EBV as well as its role in the development of cancer. EBV infection is mainly characterized by the expression of latent genes, including six nuclear antigens (EBNA1, 2, 3A, 3B, 3C, and LP) [19], two latent membrane proteins (LMP1 and 2), and EBV-encoded RNA [20]. The restricted expression of these antigens during latent EBV infection is likely a key strategy the virus utilizes to evade immune detection. In contrast, during the lytic replication phase, EBV expresses a larger array of antigens, such as BZLF1, BRLF1, BMRF1, BALF2, and BLLF1 [21]. EBV massively expresses most viral genes, which encode proteins to assemble new virus particles.

Each year, it has been estimated that EBV is the cause of approximately 2% of all global cancer fatalities [12]. EBV-linked tumors express latent antigens, and the oncogenic potential of specific latent proteins has been thoroughly investigated [22]. The prevailing view is that EBV infection triggers the activation of infected B cells, leading them into the germinal center reaction. During this reaction, the diversification machinery specific to the B cell receptor may induce further somatic mutations to aid cancer formation (e.g., MYC translocation into B cell receptor loci) [22,23,24]. More critically, certain EBV gene products confer pro-proliferative and anti-apoptotic properties on EBV-infected B cells to exert their oncogenic effects. For instance, EBV nuclear antigen 2 (EBNA2) triggers the transcription of MYC [25,26], while LMP1 induces the expression of activation-induced cytidine deaminase (AID), leading to the deregulation and translocation of *Myc*. LMP2A serves as a BCR mimic and provides survival signals to B cells within germinal centers [27]. In addition, EBV-encoded viral proteins can upregulate proteins related to cellular metabolism, promote mitochondrial activity, and produce more mitochondrial ATP. Since EBV infection of B cells is a high-energy-consuming step, enhanced production of mitochondrial ATP can promote B cell proliferation. It also accelerates the transformation of B cells by EBV, helping EBV accelerate the infection process [28].

Since there are many excellent reviews regarding the roles of EBV in cancers [29,30,31], this review will focus on exploring EBV biology, immune evasion strategies utilized by EBV, and mechanisms of triggering the onset of autoimmune diseases.

## 2. Biology of EBV

As mentioned above, EBV displays a remarkable capacity to stay dormant in numerous individuals while causing various symptoms in others [1,2]; the underlying reasons for this discrepancy are not well understood [32]. After EBV infection, there is often a prolonged incubation period, and in some instances, no symptoms may ever manifest [33]. To precisely probe into the roles of EBV in cancers and autoimmune diseases, it is important to understand the basic biology of EBV, including its viral structural components, host invasion strategies, and life cycle.

### 2.1. The Structural Components of EBV

EBV is a complex virus with a structure typical of herpesviruses [34,35]. Its genome consists of double-stranded DNA with a size of approximately 172 kilobase pairs that encodes about 85 genes [36,37]. Like other herpesviruses, EBV features an icosahedral protein capsid surrounding its double-stranded DNA core, which is composed of 162 capsomer units [38]. Beyond the nucleocapsid lies a layer of proteinaceous tegument. The entire structure is enclosed in a lipid envelope with surface projections of glycoproteins. The EBV genome encodes nine unique envelope glycoproteins (gPs) [14]. These glycoproteins determine the tropism of newly formed EBV virions, influencing their ability to invade, replicate, and persist within host cells [34]. 

### 2.2. EBV Invasion and Infection of Target Cells 

**Figure 1:**
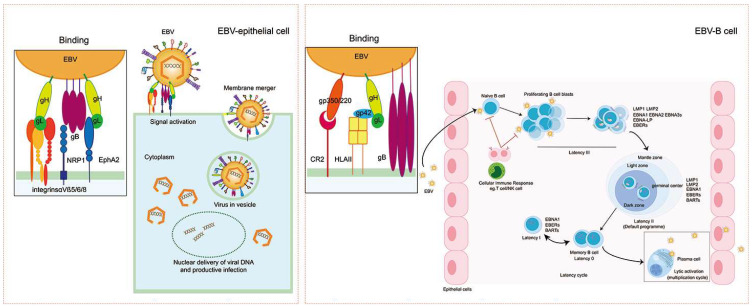
The EBV invasion mechanisms into epithelial cells versus B cells. (**Left**) EBV infection of epithelial cells: the gH/gL complex on the virus binds to the host αvβ5/6/8 integrins to attach to the epithelial cells. The interaction of EphA2 with gH/gL and gB facilitates the binding. The gH/gL complex triggers membrane fusion that can be further enhanced by the interaction of gB with NRP1 on the epithelial cells. The viral genome enters the cells and manages infection. (**Right**) EBV infection of B cells: The gp350/220 initially binds to the CR2 (CD21) on the B cell, allowing the virus to attach to the cell surface. The gH, gL, and gp42 form a complex and bind to HLA II, triggering endocytosis. At the same time, gH/gL and gB mediate the membrane fusion. After EBV infects resting naive B cells, the infected cells enter latency III. EBV-associated proteins and RNA mediate the activation of naive B cells, driving B cells to differentiate into memory B cells. Memory B cells act as reservoirs for EBV latency. In these cells, EBV gene expression is silenced to help the virus evade immune detection. However, memory B cells can be activated to differentiate into plasma cells. The plasma cells will begin lytic replication to produce infectious virus particles [48].

As mentioned above, EBV mainly infects human primary B lymphocytes and epithelial cells [15,35,39,40]. Typically, the virus attaches to the cell surface first and goes through membrane fusion to achieve infection. Additionally, EBV needs to undergo endocytosis during the infection of B cells. EBV binds to target cells through interactions between viral envelope glycoproteins and host cell receptors. EBV envelope glycoprotein 350/220 (gp350/220) binds to CD21 (or CR2) on the B cell surface [41] (Figure 1). This is followed by the formation of the complex of the viral glycoproteins gH/gL and gp42: EBV gH initially forms a complex with glycoprotein L (gL), which then interacts with soluble gp42 with extremely high affinity at a 1:1:1 ratio, forming a stable heterotrimer [42]. The gp42 can then interact with human leukocyte antigen (HLA) class II molecules on the B cell surface, triggering endocytosis. This complex also triggers membrane fusion, which can be facilitated by the direct interaction of gB with the B cell membrane [40] (Figure 1). Furthermore, EBV-infected epithelial cells express more HLA II and gp42 on the cell surface, thereby promoting EBV infection [43].

However, CD21 is not constantly expressed in epithelial cells; thus, gp350/220 plays a limited role in infecting epithelial cells [44]. Instead, the viral protein BMRF2 initially interacts with the host αVβ1 integrin to attach to epithelial cells [45]. Subsequently, the viral complex gH/gL binds to the cellular integrins αVβ5, αVβ6, and αVβ8 [46,47] (Figure 1). Following this, the cellular protein EphA2 (ephrin receptor tyrosine kinase A2) plays a pivotal role. Four extracellular domains of EphA2 participate in binding to the virus: the LBD region interacts with viral gH/gL and gB, while a cysteine-rich region and two fibronectin regions all attach to gB [47] (Figure 1). In the end, this membrane fusion process can be triggered by the interaction between gH/gL and the pre-fusion form of gB. Additionally, the interaction of gB with NRP1 on epithelial cells also facilitates membrane fusion [35].

### 2.3. The Life Cycle of EBV

As mentioned above, the life cycle of EBV encompasses primary infection, latency establishment, and lytic activation. The progression through these stages is dictated by the interactions between the virus and the host’s immune system.

#### 2.3.1. Primary EBV Infection 

So far, the prevailing view is that primary EBV infection begins in the oral cavity. Typically, primary EBV infections contracted during infancy are asymptomatic [5]; in contrast, infections acquired in adulthood often lead to mononucleosis, which is characterized by a rapid increase in the numbers of CD8^+^ T cells and NK cells that are typically important in clearing intracellular infections [49]. The underlying reasons for this discrepancy between infants and adults are unclear. Although a primary EBV infection is often asymptomatic, it can lead to a lifelong infection. 

After the initial encounter, EBV infects epithelial cells and B cells. However, no studies provide solid evidence regarding which type of cells become infected first. Studies have shown that EBV can independently infect epithelial cells in vitro, and the infected epithelial cells help the virus establish persistent infection in vivo. Moreover, there are more extensive studies focusing on B cells. EBV may first infect naive B cells or directly infect memory B cells. During the primary infection, the virus enters cells and transports the viral genome into the nucleus, where replication begins. When human B cells are infected, a variety of lytic viral genes are initially expressed along with latent EBV genes during the early or pre-latent phase of infection [50,51]. The expression of these genes activates the B lymphocytes, supports their proliferation, and helps establish latent infection. In cells with a latent infection, multiple copies of the EBV genome are consistently maintained as fully chromatinized extrachromosomal plasmids. In dividing cells, these plasmids replicate in coordination with the host cell DNA. This process helps to maintain the persistent infection with EBV. Furthermore, the virus can be reactivated, facilitating its spread to adjacent cells [52]. The primarily infected cells become the reservoir of the virus for persistent infection.

During the entire infection process of EBV, the particular antigens expressed are as follows: EBV-infected B cells express a total of nine latent antigens, which include EBNA1–6, LMP1, LMP2, and EBV-encoded RNAs [53], although EBV gene expression patterns can differ during various forms of latent infection. Although the expression of these genes can help EBV manage infection, the antigens also induce the immune response, leading to the inhibition of the infection. Among them, EBNA2, EBNA3A, EBNA3B, EBNA3C, and LMP2 can be recognized by CD8^+^ cells through the major histocompatibility complex (MHC) class I [54]. Furthermore, numerous studies have determined that NK cells contribute to controlling both primary and lytic infections [55]. 

However, host immune responses to an EBV infection can vary significantly from person to person, influenced by factors such as host genetics, environmental conditions, and the individual’s age [56]. For instance, immune co-stimulatory proteins such as CD27 and SLAM family members, along with the co-inhibitory CTLA-4 receptor, regulate the immune responses to impact the course and outcomes of EBV infections [57]. 

#### 2.3.2. EBV Latency 

Latency is the state of a persistent viral infection without producing new virus particles. EBV mainly resides within the memory B cell compartment, though it might also be found in epithelial cells [2]. Moreover, EBV mainly establishes latent infection and latent phase in B cells. The latency phase is characterized by the expression of specific viral latent genes. Among these, six genes encode the nuclear proteins known as EBNA, three encode the membrane protein LMP, and two encode the non-coding RNAs [35]. The profiling of gene expression patterns in various cell lines, such as Burkitt’s tumors and EBV-immortalized lymphoblastoid cell lines, has revealed the existence of at least three distinct latent phases throughout the life cycle of EBV [2]. Each latent phase is characterized by unique viral gene expression patterns: latency 0/I, where only EBV nuclear antigen 1 (EBNA1) is expressed; latency II, where only EBNA1, LMP1, and LMP2 are expressed; and latency III, where all latent viral proteins, including six EBV nuclear antigens (EBNAs) and three latent membrane proteins (LMPs), are expressed [33].

After the primary infection, EBV-infected naive B cells enter latency III. During this phase, the cells express all latent proteins and undergo the viral growth program, driving the naive B cells to become proliferating blasts. The cytotoxic T cells (CTL) are recruited to recognize epitopes from latent proteins and destroy the infected cells. The growth program may help the cells establish latent infection by evading the CTL response. EBV utilizes its growth program to stimulate newly infected B cells, helping their differentiation into resting memory B cells [58]. The viral genome activates naive B cells from EBV latency III and drives the cells to enter the germinal center (GC) program [59]. Due to different extents of transcriptional silencing, only three latency-related proteins (EBNA1, LMP1, and LMP2) can be found in centroblasts and centrocytes [60], marking this period as latency II (also referred to as the default program). In latency II, latent proteins help EBV-infected B cells to survive in the germinal center, begin cell proliferation, and drive the naive B cells to differentiate into memory B cells. These cells are evenly distributed between the memory compartments of Waldeyer’s ring and the peripheral blood [61]. Latency II is mainly found in EBV-associated Hodgkin lymphomas and nasopharyngeal carcinomas (NPCs). Memory cells that have been latently infected undergo a shutdown in the expression of viral proteins and enter the latency program [62]. 

Latency I/0 is present in memory B cells [63]. After EBV-infected germinal center B cells migrate to the peripheral blood, a stable reservoir of resting, viral-genome-positive memory B cells is established. At this phase, almost all viral antigen expressions are suppressed, and this state is known as latency 0 [62,64]. However, EBNA1 is transiently expressed during memory B cell division, driving the cells to enter latency I from latency 0 [62]. The viral genome is passed on to daughter memory B cells. These memory cells provide an ideal niche for the virus, allowing it to persist for extended periods as memory B cells infrequently die and express no viral proteins detectable by the immune system. Furthermore, in resting state cells, the virus does not pose a threat to its host as the growth-promoting genes are no longer expressed [2].

Furthermore, the EBV in memory B cells at latency 0/I can be reactivated, and the virus can drive the cells to enter the lytic cycle to produce new virus particles [65].

#### 2.3.3. Lytic Replication of EBV

As mentioned above, EBV is causally linked to several types of cancer [10]. Even though EBV tumors consist of cells in various latency programs, numerous studies have indicated that the lytic cycle also plays an important role in the oncogenic ability of EBV. The lytic cycle is initiated to allow cells to express most viral genes and assemble new virus particles, leading to a high viral load [66,67]. 

The lytic cycle of EBV consists of three sequential phases: the immediate early phase, the early phase, and the late phase. The immediate early phase and early phase are collectively referred to as the early stage. To be more specific, the stage that mediates viral DNA amplification is classified as “early stage”, while the stage that synthesizes viral proteins and conducts viral particle assembly is defined as “late phase”. The early stage is further divided into two phases. The stage that initially mediates the transcription of viral DNA replication genes is defined as the “immediate early phase”. The remaining part is known as the “early phase”. The first genes to be transcribed during the immediate early phase are BZLF1 and BRLF1, which encode the transcription factors ZEBRA (ZTA) and RTA, respectively. These proteins, ZEBRA and RTA, activate the promoters of early genes. In the early phase, the activation of the early genes encodes the viral replication proteins (including the viral DNA polymerase, its processivity factor, thymidine kinase, and the helicase-primase complex) and proteins involved in the expression of late genes. Subsequently, the late phase starts, and late genes are expressed to encode structural proteins such as the viral capsid antigen (VCA), the viral protease important for capsid maturation, and the envelope glycoproteins. Collectively, these components allow for the assembly of new infectious viral particles [68].

BZLF1 and BRLF1 promoters are initially activated by cellular transcription factors. Subsequently, ZEBRA and RTA can activate their own promoters and each other’s promoters. ZTA-response elements (ZREs) in the EBV genome usually contain CpG motifs that are methylated in latently infected cells. Although methylation inhibits the activation of transcription factors, ZEBRA prefers binding to methylated promoters. ZEBRA’s ability to activate methylation promoters facilitates the rapid reactivation of EBV. Therefore, methylation of the EBV genome plays a critical role in lytic activation [50,69,70]. 

The promoters are initially activated by transcription factors to start the lytic cycle. Studies have shown that BCR stimulation and plasma cell differentiation may be responsible for the trigger of lytic activation. The proteins encoded during BCR stimulation and plasma cell differentiation can activate the BZLF1 and BRLF1 promoters [68]. Moreover, studies also found that xenobiotics, including tobacco smoke, pollution, and pesticides, can promote the activation of the lytic phase of EBV, contributing to the development of lymphoid and epithelial cancers [39].

## 3. Major EBV-Encoded Antigens 

### 3.1. LMP1

Latent membrane protein 1 (LMP1), the first discovered latent gene of EBV, has demonstrated its ability to transform cell lines and change cell phenotypes due to its oncogenic potential [71,72]. LMP1 has three distinct functional domains in the C-terminal region (amino acids 187–386): C-terminal activation regions 1, 2, and 3 (CTAR1, CTAR2, and CTAR3). CTAR1 (amino acids 194–231) has been shown to initiate cell proliferation, while CTAR2 (amino acids 351–386) promotes the permanent growth of lymphoid cell lineages [73,74]. CTAR3 is an additional region between the CTAR1 and CTAR2 regions, which mediates the function of LMP1 to induce sumoylation and enhances the ability to induce cell migration [75].

LMP1 is a crucial oncogene of EBV and mainly regulates cellular signaling pathways, which trigger the activation of cell proliferation and anti-apoptotic programs. In addition, LMP1 also possesses a diverse range of functions, including induction of cytokines and chemokines expression, immune modulation, widespread alteration of gene and microRNA expression patterns, and regulation of tumor angiogenesis [76].

In murine models, LMP1 can trigger T cell responses to tumor-associated antigens (TAAs) [23,24]. LMP1 can regulate corresponding signaling pathways to increase the expression of several TAAs across various cell types. These antigens are presented on major histocompatibility complex class I (MHC-I) and class II (MHC-II) molecules on the surface of B cells to activate the cellular immune response. LMP1 also functions like a perpetually active tumor necrosis factor receptor, emulating CD40. However, it can activate multiple signaling pathways without a ligand [53,74]. For instance, CD40 and LMP1 share similar signal transduction through activating the NF-*κ*B pathway, and hindering the NF-*κ*B pathway in lymphoblastoid cell lines precipitates spontaneous cell apoptosis [77,78].

In LMP1-related research, a gene known as Ugene (FAM72A) was discovered. When LMP1 was transferred into NPC cells, a higher expression of Ugene could be induced. Hence, Ugene is also referred to as an LMP1-induced protein (LMPIP). Research on NPC has demonstrated that LMPIP is a key mediator of LMP1, contributing to decreased cellular ROS generation and accelerating the G1/S transition via the mitogen-activated protein kinase (extracellular signal-regulated kinase 1/2, MAPK1/2) and NF-*κ*B signaling pathways [79]. Moreover, like LMP1-transfected cells, cells with overexpressed LMPIP demonstrated increased expression of several proteins, particularly CDK4, cyclin D1, cyclin B1, and E2F1 [80,81,82]. These data suggest that LMPIP could play a significant role in LMP1-induced tumorigenesis [71], although the underlying mechanisms are still unclear.

### 3.2. LMP2

There are two isoforms of LMP2, namely LMP2A and LMP2B [83]. The structural difference between these two isoforms is that LMP2B has 119 fewer amino acids at the N-terminus than LMP2A [27]. LMP2, especially LMP2A, plays an important role in the latency phase of EBV, and LMP2A exhibits a higher mRNA expression level than LMP2B in 98% of NPC cases [84]. LMP2A effectively maintains the viral latency by enhancing the survival of infected host cells, while LMP2B exhibits a regulatory effect on the distribution and function of LMP2A [27,85].

The N-terminal cytoplasmic domain of LMP2A contains many tyrosine residues, constituting the immunoreceptor tyrosine-based activation motif (ITAM) [86]. The ITAM motif of LMP2A (amino acids 74–88, including tyrosine residues Y74 and Y85) has a similar function as the ITAM in the B cell receptor (BCR) signaling complex [87]. This ITAM motif recruits the SH2 domain that contains signaling proteins, such as Syk kinase, leading to the recruitment of multiple linkers (SLP-65, CrkL, Vav, and Shb), which in turn recruit and activate PI3 kinase/Akt and other downstream signaling molecules [88,89,90]. Although LMP2A acts as a functional BCR mimic, it remains to be investigated whether LMP2A can compete with host membrane-bound BCR for antigen recognition. In addition to regulating BCR signaling, other studies have shown that LMP2A can regulate the production of IL-10, which could inhibit the host antiviral immune responses while promoting the survival of B-cell lymphoma [91,92]. 

However, LMP2A-mediated signaling can lead to two opposite functional outcomes in BCR: acting as a BCR mimic to activate BCR signaling or depriving the BCR signaling complex of its components to inhibit BCR signaling. It is still unclear in which contexts LMP2A will exhibit activating versus inhibitory effects on BCR signaling [27,93], although it has been postulated that this may depend on the expression level of LMP2A. BCR signaling can activate several transcription factors to enhance certain activities of B cells (e.g., cell proliferation), and low-level LMP2A can enhance the signaling outcome of BCR cross-linking, helping B cells survive in germinal centers and mature into memory B cells. In contrast, abnormally high levels of LMP2A may restrain BCR signal transduction [94]. In addition to the above-mentioned reasons where LMP2A simulates BCR activation by providing a constant low-level signal, leading to feedback inhibition of the normal BCR pathway and weakening the B cell response to actual antigen binding, other immunomodulatory mechanisms include the inhibition of Syk kinase activation and the PI3K/Akt pathway, which reduces the sensitivity of B cells to BCR signaling [88,89,90,94]. 

### 3.3. EBNA1

EBNA1 is the only EBV protein found in all EBV-related malignancies, offering opportunities for targeted therapeutic intervention [95,96]. EBNA1 is essential for viral genome maintenance and controlling viral gene expression in different life cycles [97]. The structural particularity of EBNA1 determines its important role in viral genome replication. It usually exists as a homodimer, passing the viral genome connected with the C-terminus to the cell chromosome connected with the N-terminus to help complete replication [95,98].

Tempera et al. analyzed EBNA1 ChIP-Seq data combined with shRNA in vitro experimental data and identified genes essential for B cell survival, such as IL6R, KDMC4C, EBF1, and MEF2b, which are directly and positively regulated by EBNA1 [99]. EBNA1 can also trigger the occurrence of epithelial cancers and B-cell lymphoma. Don Cleveland’s team revealed that EBNA1 bound a cluster of EBV-like sequences at a fragile site on human chromosome 11, where increased protein abundance would trigger chromosome breakage. The research team also examined the whole genome sequencing data of 2439 cancer samples of 38 cancer types and found that EBV-infected tumors showed higher levels of chromosome 11 abnormalities, especially nasopharyngeal carcinoma [96,100]. Some studies have also shown that EBNA1 can inhibit the transcription of the p53 gene, which is known as the “genome Guardian” [101,102]. In addition to EBNA1, it is proven that other nuclear antigens, including EBNA2, EBNA-3A, EBNA-3C, and EBNA-LP, are also essential for the in vitro transformation of primary B cells into lymphoid cell lineages or the survival of host cells [103,104,105,106].

However, EBNA1 is highly immunogenic, so T cells generated against EBNA1 can be detected in infected individuals. Therefore, the virus seems to have evolved a strategy to utilize nucleolin, a host cytokine, to bind to the RNA G-quadruplex within EBNA1 mRNA to limit its expression and avoid immune recognition [107]. Furthermore, EBNA1 can interact with host cell proteins and regulate the activities of cellular pathways that promote viral persistence and the survival of infected host cells. EBNA1 also promotes the development of cancers and facilitates the transformation of B cells.

Although EBV can help infected B cells to finish maturation and drive B cells into memory cells, EBV-induced memory B cells in the absence of host BCR recognizing the corresponding antigens exhibit significant defects in antibody production, such as impaired antibody binding affinities and reduced antibody switch recombination. As mentioned, LMP1 can function as the CD40 mimic, inducing the B cells to secrete BAFF and APRIL. These cytokines will strengthen antibody class switch recombination, resulting in an unbalanced proportion of IgA, IgG, and IgM. LMP1 also affects the activity of activation-induced cytidine deaminase, which has an impact on antibody production [108]. In EBV-infected B cells, antibody production is significantly elevated, and most of the antibodies produced by EBV-infected plasma cells are IgM [109,110]. Some of these IgM can cause damage to host tissues, and damaged tissues/cells may be engulfed by antigen-presenting cells, with tissue antigens being presented to T cells that activate CD40 signaling, inducing B cells to produce high-affinity IgG antibodies that facilitate the development and exacerbation of autoimmune diseases [108]. Moreover, it seems likely that LMP2A can protect autoreactive cells from either decreased half-life or autoantigen-induced anergy [94].

## 4. Interaction between the Host Immune System and EBV

The human host has developed a range of innate and adaptive immune strategies to effectively fight against pathogens or cancerous cells that are derived from infections [111]. As mentioned above, EBV can establish lifelong persistence in the infected host despite its powerful host immune defense capacity. EBV’s ability to achieve successful lifelong persistence demonstrates its evolved, effective strategies and mechanisms, which aim to exploit, diminish, or nullify the host’s immune responses to ensure its survival. In this section, we will introduce the host immune responses against EBV as well as the evasion strategies the virus adopts for its benefits.

### 4.1. Host Innate Immune Responses to EBV 

EBV can coexist with the human body for a long time after infection, clearly indicating that the virus has adapted to the host’s innate immunity during its co-evolution with humans. Innate immunity is the body’s first line of defense against pathogens, activating within hours of an antigen’s appearance [112]. These mechanisms include physical barriers such as skin, chemicals in the blood, and innate immune cells that engulf and digest foreign non-self-pathogens [113].

Epithelial cells constitute the first innate barrier to microbial infections. Innate immune cells also utilize a limited number of germline-encoded pattern-recognition receptors (PRRs) to detect a diverse array of pathogen-associated molecular patterns (PAMPs). The expression levels of PRRs significantly vary among different immune cell subsets [114]. Numerous studies have demonstrated that EBV products act as PAMPs to stimulate the host cell PRRs, and multiple PRR signaling pathways are activated to initiate an innate antiviral immune response during EBV infection [115,116]. This response triggers the production and release of various cytokines, including interleukins, tumor necrosis factor, and interferons. These cytokines, in turn, trigger apoptosis and inflammatory processes to create an unfavorable environment for viral replication [117]. The innate immune response can also help the host strengthen the adaptive immune response. For example, as one of the most important PRR signaling pathways, TLR9 signaling plays a central role in the host's innate defense against viruses. TLR9 can specifically bind to unmethylated CpG DNA sequences and distinguish bacterial DNA from host DNA [118,119]. The deficiency of the TLR9 signaling pathway can result in extensive infection of immune cells (e.g., macrophages), leading to a low efficiency of viral clearance [120]. EBV genomic DNA activates TLR9 signaling in B cells and ultimately leads to the activation of NF-*κ*B, which in turn promotes the production of pro-inflammatory cytokines and stimulates the activation and proliferation of B cells [121]. However, there is no clear quantitative analysis of the extent of EBV clearance contributed by TLR9 signaling.

### 4.2. Host Adaptive Immune Response to EBV 

Adaptive antiviral immunity relies on robust, memory-based responses from virus-specific T and B lymphocytes. Cellular immunity (involving CD4^+^ T cells and CD8^+^ T cells) and humoral immunity (involving antibodies) play important roles in controlling both the initial [122] and persistent [53] stages of viral infection. Typically, the detection and elimination of EBV-infected cells depend on cellular immunity. T cells can recognize virus-derived peptides presented in complexes with MHC molecules to attack infected cells [123]. In individuals with established EBV infections, the T cell response demonstrates substantial frequencies of EBV-specific CD8^+^ and CD4^+^ T cells, and more severe diseases caused by EBV infection were observed in individuals with impaired T cell development or function [124]. 

Humoral immune responses begin with the direct engagement of antigenic substances by B cell receptors. These responses require support from T lymphocytes and specific cytokines to facilitate antibody class switching and a refined antibody binding affinity. Research into humoral immune responses to EBV has been conducted with diagnostic, pathogenic, and protective (vaccination) objectives [34,125].

Overall, humoral immunity mainly plays a role in the late stage of EBV infection to control the virus’s spread, while cellular immunity is crucial for controlling both the primary and sustained stages of EBV transmission. This is because EBV triggers robust cellular immune reactions to certain proteins during its lytic cycle. During primary EBV infection, both IgM and emerging IgG reactions to nucleocapsid and envelope proteins can be detected. Furthermore, IgG reactions to certain immediate early and early lytic cycle proteins, along with the latent proteins EBNA1 and EBNA2, are often observed. Cytotoxic T lymphocytes are a major determinant in controlling acute EBV infection [126,127] and directly target both lytic and latent EBV antigens [128]. During acute infection, more than 44% of CD8^+^ T cells target a single lytic EBV epitope. The majority of these epitope-specific cells display an activated/memory phenotype [129]. In contrast, late in infection, epitope-specific CD8^+^ T cells show an increased frequency of targeting latent EBV proteins, confirming that cytotoxic T lymphocytes are the most important cells limiting infection during the recovery period of viral infection [129].

### 4.3. Immune Evasion of EBV 

Although the human body possesses a strong immune system capable of controlling EBV infection, it is challenging to completely eliminate the virus [130]. Like other herpesviruses, EBV persists in infected individuals by balancing viral replication and host antiviral immunity [16].

In response to the robust host immune response, EBV has developed various strategies to evade the host’s innate and adaptive immunity. A significant portion of EBV-encoded proteins and non-coding RNAs are dedicated to facilitating immune evasion [131,132,133]. To establish infection and maintain long-term coexistence with the body, EBV manages to modulate the corresponding signaling pathways to minimize their antiviral activity. For the innate immune response, since PRR signaling leads to activation of the transcription factors interferon regulatory factors (IRF) 3 and 7 [134] or NFκB [135], creating an environment that is inhospitable to the virus, EBV can inhibit PRR signaling to restrain the activation of IRF and NF-κB, such as TLR signaling, cGAS-STING signaling, and RIG-I-MAVS signaling. Additionally, EBV can directly suppress the expression of IRF or the activation of the NF-κB pathway to evade innate immunity. By regulating specific molecules, EBV can inhibit or block one or more of these pathways, helping the virus escape innate immunity.

**Figure 2:**
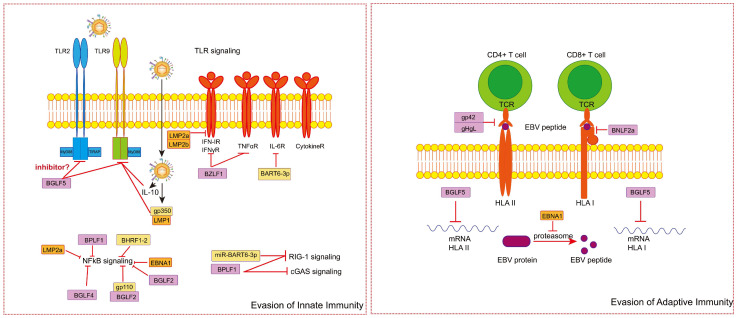
The mechanisms of the immune evasion of EBV. (**Left**) In the evasion of innate immunity, EBV can modulate relevant molecules to inhibit PRR signaling and suppress the activation of IRF and NF-κB. EBV can also directly act on and inhibit IRF or NF-κB signaling pathways. All regulations ultimately reduce the release of inflammatory cytokines, helping EBV survive in cells. (**Right**) In the evasion of adaptive immunity, EBV mainly blocks the activation of CD8^+^ and CD4^+^ T cells by disrupting the HLA and TCR binding pathways. EBV can reduce the presentation of EBV peptides and block the combination of HLA and TCR, resulting in T cells being unable to be activated and inhibiting adaptive immunity.

Next, we will provide a detailed introduction to several signaling pathways involved in EBV immune evasion and their roles in this process:

I. The inhibition of the PRR signaling pathway
  (1) The TLR signaling pathway
    TLR signaling is the most widely studied PRR signaling pathway. Overexpression of EBV latent protein LMP1, which is encoded by EBV, can reduce the activity of the TLR9 promoter and decrease the expression of TLR9 mRNA and protein in B cells [121,122]. LMP1 also interacts with the lytic phase protein gp350 to downregulate the expression of TLR9 [136]. EBV alkaline exonuclease BGLF5 can degrade TLR9 mRNA to inhibit its expression [137]. BGLF5 also decreases the expression of TLR2 [5]. Through these mechanisms, EBV may avoid TLR signaling, which can induce cell death and inhibit lytic reactivation [123,124] (Figure 2).
  (2) The RIG-I-MAVS pathway
    The RIG-I-like receptor family is also an important member of PRRs, which can recognize viral RNA or replication transcripts in the cytoplasm and trigger innate immunity. However, miR-BART6-3p, a microRNA encoded by EBV, can downregulate RIG-I expression. This microRNA also targets the 3′UTR of RIG-I mRNA to inhibit RIG-I signaling and the host immune response. At the same time, BART6-3p can also inhibit IL-6 receptor expression, though it is not verified if it further inhibits NF-κB downstream signaling. Moreover, EBV deubiquitinase BPLF1 can deubiquitinate TBK1 in RIG-I-MAVS signaling to inhibit the pathway [138] (Figure 2).
  (3) The cGAS—STING pathway
    The EBV deubiquitinase BPLF1 can deubiquitinate STING to inhibit the cGAS-STING pathway [138] (Figure 2). BLRF2, the tegument protein of EBV, blocks the production of cGAMP by binding cGAS and inhibiting its activity [139]. EBV also upregulates tripartite motif-containing protein 29 (TRIM29) to ubiquitinate STING. The degradation of STING leads to the inhibition of the cGAS—STING pathway [140].
II. The inhibition of the expression of IRF

EBV can directly inhibit the activation of IRF. For example, the latent proteins LMP2A and LMP2B can affect the stability of IFN receptors by inducing ubiquitination of IFN receptors to suppress the activation of IRF. The immediate early EBV transactivator BZLF1 engages with IRF7 to impede its transcriptional activity at the IFNα4 and IFNβ promoters, restraining the function of IRF [141]. In addition, BZLF1 can downregulate TNF-R1 promoter activity, reducing TNF-R1 expression, weakening the TNF-α response, and inhibiting infected cell apoptosis (Figure 2).

III. The inhibition of the activation of the NF-κB pathway

EBV can also directly inhibit the activation of the NF-κB pathway. One of the mechanisms is that EBNA1, gp110, and BGLF2 can inhibit the activation of NF-κB by reducing the phosphorylation and nuclear translocation of p65 in the NF-κB complex. However, EBNA1 inhibits the phosphorylation and nuclear translocation of p65 by reducing the phosphorylation of IKK α/β. The glycoprotein gp110 interacts with IKKi and restrains its K63-linked polyubiquitination. BGLF2 interacts with NF-κB subunits p65 and p50 and inhibits the phosphorylation of p65 Ser536 to prevent nuclear translocation [142]. BHRF1-2 miRNA, a microRNA encoded by EBV, blocks the IL-1 pathway to inhibit the activation of NF-κB. Herpesvirus kinase BGLF4 phosphorylates the Thr3 site of the coactivator UXT, destroys the interaction between NF-κB and UXT, and downregulates the transcriptional activation of NF-κB [143]. The EBV deubiquitinase BPLF1 deubiquitinates TRAF6 and restrains TRAF6-mediated NF-κB signal transduction [144] (Figure 2).

Following the initial innate immune response, EBV infection also triggers potent T cell responses specific to the virus, targeting epitopes derived from both the lytic and latent phases of EBV. A crucial element of EBV’s immune evasion strategy involves its capacity to enter a latent state, characterized by minimal expression of viral genes and limited presentation of viral peptides to the immune system [49,145]. However, EBV can also hinder the activation of both CD8^+^ and CD4^+^ T cells by disrupting different stages of the HLA class I and class II antigen presentation pathways. For example, the EBV alkaline exonuclease BGLF5 degrades HLA class I mRNAs, thus diminishing antigen presentation [146,147]. EBNA1 can interfere with the degradation of EBV protein, inhibit the production of peptides, and reduce antigen presentation [148] (Figure 2). Moreover, EBV gp42 can bind to HLA II and block the interaction of HLA II with TCR, preventing the activation of CD4^+^ T cells, and the gH/gL complex can stabilize and promote the expression of gp42 [148]. Additionally, BNLF2a, encoded by the early EBV gene, blocks ATP binding to the transporter associated with antigen processing (TAP), leading to the loss of the function of TAP. This results in TAP being unable to transport the viral peptide, thereby inhibiting the binding of TCR to HLA [149] (Figure 2). Although EBV interferes with antigen presentation in various ways, it does not infect all antigen-presenting cells equally. Although monocytes, dendritic cells, and macrophages can also be infected by EBV [150,151,152], the infection efficiency is lower than that of B cells.

Given EBV’s lifelong presence in the host and its continuous coexistence, the virus likely gains no benefit from damaging the host’s cells. Thus, EBV is assumed to delicately manipulate the host's innate immunity, maintaining a finely tuned balance rather than completely suppressing signaling pathways. The modulation of innate responses by EBV not only leads to partial immune evasion but also promotes its own growth and transformation functions [130,153,154]. For example, it has been reported that the BORF2 protein, encoded by EBV, maintains the integrity of the viral genome by inhibiting APOBEC3B in cells [155]. However, EBV immune evasion may also contribute to the occurrence of malignant tumors. EBV has numerous strategies to evade the body’s immune regulation, and many specific mechanisms still require further experimental elucidation. Research into the immune escape mechanism of EBV can provide a robust theoretical basis for the clinical prevention and treatment of EBV.

## 5. EBV and Autoimmune Diseases 

In addition to cancers, EBV has been linked with a range of autoimmune diseases [4,14,156]. In this section, we will focus on the roles of EBV in three widely researched autoimmune diseases: systemic lupus erythematosus, multiple sclerosis, and rheumatoid arthritis.

### 5.1. EBV and Systemic Lupus Erythematosus 

Systemic lupus erythematosus (SLE) is a multifaceted systemic autoimmune disease characterized by the presence of autoantibodies towards nuclear antigens (ANAs) in almost all SLE patients. SLE patients have major symptoms such as inflammation, rash, and vascular lesions, and many patients also suffer from systemic pain and fatigue [157]. The exact cause of SLE is still unclear. However, the dysregulation of the immune system is a critical factor in developing SLE. The etiology of SLE is multifactorial, including genetic and environmental factors [158]. 

Many viruses can trigger autoimmune responses against host nuclear antigens in SLE patients. For example, cytomegalovirus and human herpesvirus 6 can cause immune activation and may promote autoimmunity through molecular mimicry [159,160]; hepatitis C virus infection can lead to persistent immune activation and the production of autoantibodies, potentially leading to the development of autoimmune diseases [161]. 

The association between SLE and EBV infection was first reported in 1969 [162]. Individuals who contract EBV later in life may have a higher risk of developing SLE due to the intense immune response [163]. The number of EBV-infected B cells increases in the peripheral blood of SLE patients, and these cells express latent and lytic genes [4]. For example, the increased expression level of BZLF1, which is responsible for triggering the lytic cycle of EBV, has been detected in SLE patients [4]. In addition, primary infection with EBV causes an IgG response to viral capsid antigen (VCA). When EBV is reactivated in vivo, it causes an IgG response to the early antigen (EA). In SLE patients, EA-specific IgG levels are detectable and VCA-specific IgG levels are elevated, indicating that EBV is reactivated [164]. Moreover, EBV produces three latent membrane proteins (LMP1, LMP2A, and LMP2B) that emulate the signals necessary to sustain normal B cell differentiation, even without T cell help. 

Interestingly, the first lupus-specific autoantibodies came from specific antibodies against EBNA1. In EBV-immortalized B cells, almost half of the SLE risk alleles can be occupied by EBNA-2. EBNA-2 is involved in the formation of transcriptional complexes at risk loci. Host transcription factors bind to SLE risk loci only in the presence of EBV [165]. This suggests that EBV can act as an environmental risk factor, shaping the environment needed for SLE to develop, and also as a genetic factor.

Molecular mimicry is widely utilized to explain the pathological mechanism of EBV in SLE. The molecular resemblance between SLE autoantigens and EBV antigens might give rise to an autoimmune response [158]. Due to structural similarities, antibodies to viral antigens like EBNA-1 can cross-react with autoantigens [166]. Although the most common autoantibody in SLE patients is the autoantibody to nuclear antigens, other antibodies, including anti-Sm, anti-Ro, and anti-dsDNA, are also present in many SLE patients. Molecular mimicry exists between EBNA1 and at least three antigens in SLE (SmB/B’, SmD, and 60 kD Ro) [167,168]. EBNA1 is homologous to SmB/B’ and SmD. In SLE, autoantibodies targeting SmB/B’ and SmD epitopes can cross-react with different domains of EBNA1 [169,170]. In addition, the anti-Ro antibody, the earliest detectable autoantibody in some SLE patients, can cross-react with EBNA1 [166]. EBNA1 drives the production of anti-dsDNA, anti-Sm, and anti-Ro antibodies, enhancing the autoimmune response in SLE. When the self-protein binds to the B cell receptor, it is internalized, processed, and then presented to T cells. This autoimmune response can be further diversified through a process known as epitope spreading, where B cells can internalize and present peptides from the entire protein containing the cross-reactive epitope to T cells. Consequently, this assists the antibody response towards other epitopes on the protein by B cells [166,171,172]. EBV can also facilitate epitope spreading, expanding the presentation of other antigenic determinants of lupus-associated autoantigens. This also exacerbates the autoimmune response in SLE [173,174]. 

Additionally, EBV interleukin-10 (vIL-10) is a homolog of human IL-10 (hIL-10). Due to its high homology, vIL-10 competes with hIL-10 for receptors, restrains hIL-10 signaling, and inhibits the inhibitory effect of hIL-10 on immune responses. The viral homolog vIL-10 also induces a pro-inflammatory phenotype in monocytes and reduces the ability to clear apoptotic cells [175]. These enhance antigen presentation and the inflammatory response, leading to the production of autoantibodies, which exacerbate SLE. 

In addition to being a functional homolog of CD40, LMP1 stimulates B cells to produce IFN-α, IL-6, IL-10, B cell-activating factor (BAFF), and a proliferation-inducing ligand (APRIL) [59]. BAFF and APRIL facilitate the survival of B cells, including autoimmune B cells, and T cell-independent antibody production [176,177]. Elevated IFN-α in SLE patients is associated with higher disease activity, which leads to enhanced antibody production and tissue damage. Additionally, LMP1 can induce class switch recombination by inducing abnormal BAFF and APRIL expression in B cells, leading to the increased production of IgG, IgA, and IgE, which contribute to the pathogenesis of SLE [177].

Overall, EBV can influence immune responses through a variety of mechanisms, including the creation of cross-reactive antibodies [178], secretion of IFNα [179], antigen-independent B cell activation, alteration of gene expression, and suppression of anti-inflammatory responses [180]. Evidence in SLE patients points to an increased reactivation of EBV, possibly arising from a combination of dysregulated immune responses and genetic risk factors. In addition, viral homologs like vIL-10 can modify the immune response in a way that may worsen the autoimmune reaction in genetically prone individuals [175].

### 5.2. EBV and Multiple Sclerosis 

Multiple sclerosis (MS) is a chronic autoimmune disease that affects the central nervous system (CNS), which includes the brain and spinal cord. It is characterized by the immune system mistakenly attacking the protective sheath (myelin) that covers nerve fibers, causing communication defects between the brain and the rest of the body. There are several types of MS, including relapsing–remitting MS, secondary progressive MS, and primary progressive MS [181,182]. The causes of MS are multifaceted and not fully understood. The disease’s etiology includes a combination of environmental factors along with a multitude of genes that may moderately heighten the risk of developing MS [183,184]. 

Epidemiological studies have shown a correlation between EBV infection and MS onset [185,186]. A substantial body of research indicates that infection with EBV might be a critical co-factor or even a trigger interplaying with other human herpes viruses and human endogenous retroviruses, and possibly even a prerequisite for the onset of autoimmune diseases [13,187,188]. For instance, the MS risk notably increases after infectious mononucleosis, which is primarily caused by EBV infection [189]. The remarkable similarity between the epidemiology of MS and IM also supports the correlation between EBV infection and MS [183]. Furthermore, the elevated serum titers of anti-EBNA antibodies in MS conditions and the presence of EBV in demyelinated lesions of some MS patients indicate the causality of EBV to MS [190,191]. Other immunological evidence includes the presence of EBV-reactive oligoclonal bands in the CSF of MS patients, the deficiency of cytotoxic T lymphocytes in MS conditions, and the interactions between MS risk alleles and EBNA2, in which EBNA2 contributes to the enrichment of MS risk alleles for transcription and the MS risk alleles facilitate EBNA2 to promote B cell immortalization [165,192,193]. A longitudinal analysis involving 10 million young adults reveals a 32-fold increased risk of MS after EBV seroconversion and provides definitive epidemiological evidence of EBV as the leading cause of MS [13], although the exact mechanisms are unclear. Meanwhile, numerous studies have found an association between a later age of EBV infection and an increased risk of developing MS [194,195]. The risk of both infectious mononucleosis and MS increases when primary EBV infection occurs after the age of 10 years. At this age, thymic negative selection of autoreactive T cells slows, and T helper 1 cell-mediated responses peak [195]. There are still many gaps in our understanding of how immune system maturation triggers EBV-driven autoimmune reactivity, leading to the development of MS.

Chronic latent and persistent infections continuously provide viral antigenic triggers. The immortalization of EBV-infected B cells, together with the bypassing of negative selection and the requirement for T cell help through the virally encoded proteins LMP1 and LMP2, contributes to the increased risk of continuous autoreactive antibody production [3,162]. In MS, autoreactive antibodies have been found to cross-react with viral proteins, particularly with EBNA1 [196]. Analyses conducted with peptide libraries have identified multiple domains within EBNA1 that are specifically recognized by autoreactive immune responses. The peptide consisting of amino acids 391–410 in EBNA1 mimics the sequence found in host CRYAB (alpha-crystallin B chain) amino acids 1–15, with an overlapping sequence of RRPFF [196]. Another study demonstrated the molecular mimicry between EBNA1 domain amino acids 386–405 and the glial cell adhesion molecule (GlialCAM), which is a CNS protein that plays a role in maintaining the integrity and functioning of the myelin sheath. Cross-reactive antibodies against both EBNA1 and GlialCAM are prevalent among MS patients [197]. 

**Figure 3:**
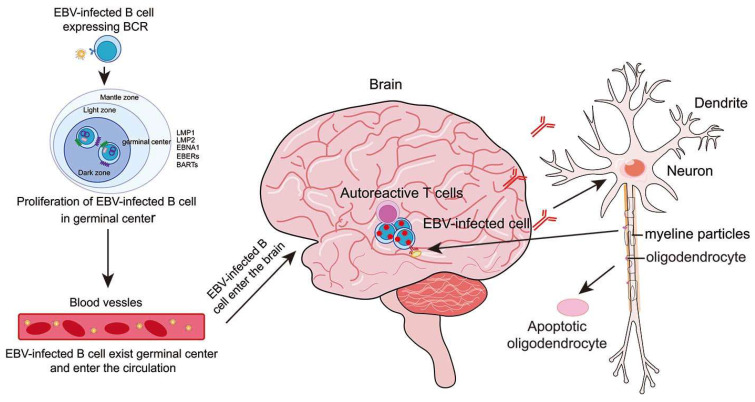
The mechanisms of EBV in the pathogenesis of multiple sclerosis. After EBV infection with B cells, B cells enter the germinal center program to proliferate. After proliferation, infected B cells leave the lymph nodes and enter the bloodstream. The infected cells follow the circulation into the brain and stay in the brain cells. Autoreactive T cells in the CNS are activated by contact with infected B cells. In addition, dendritic cells that present and release viral antigens also contribute to activation. The low efficiency of removing infected B cells results in their accumulation in the CNS. EBV-infected B cells in the CNS produce autoantibodies that act on the myelin sheath, leading to the damage of the myelin sheath and releasing myelin sheath and oligodendrocyte debris. EBV-infected B cells also act as powerful antigen-presenting cells to stimulate autoreactive T cells to recognize and attack myelin, contributing to inflammation of the CNS. The B cells and T cells in the CNS play their function to damage other cells and cause autoimmune diseases.

In addition to antibodies, CD4^+^ T cells are also involved in cross-recognition between EBV peptides and host molecules. The crystal structure of the DRB5*0101-EBV peptide complex revealed a marked structural mimicry of the DRB1*1501-restricted myelin basic protein (MBP), both of which can be recognized by the patient-derived autoreactive TCR [198]. EBV DNA polymerase peptide EBV 627–641-specific CD4^+^ T cells can cross-recognize an immunodominant myelin basic protein peptide, MBP 85–99 [199]. The viral lytic proteins BHRF1 and BPLF1 are cross-reactive with the self-protein RASGRP2, which is targeted by autoreactive CD4^+^ T cells [200] (Figure 3).

### 5.3. EBV and Rheumatoid Arthritis

Rheumatoid arthritis (RA) is a multifaceted autoimmune condition characterized by persistent joint inflammation, resulting in progressive joint deterioration, functional impairment, and a substantial global healthcare burden [201]. RA manifests a diverse array of symptoms, including joint pain, stiffness, swelling, and fatigue, which significantly affect patients’ overall quality of life [202]. This debilitating condition affects approximately 1% of the global population, with a striking predilection for women [203,204]. Despite decades of research and advances in treatment strategies, the exact etiology of RA remains elusive, with multifactorial influences, including genetic predisposition and environmental factors, contributing to disease susceptibility and progression [205,206]. 

In recent years, there has been growing interest in the potential role of infectious agents, particularly the Epstein–Barr virus (EBV), in the pathogenesis of RA, stimulating investigations into the intricate relationship between viral infections and the development of autoimmune diseases [207,208]. The possible implication of EBV in RA was first described by Alspaugh et al., who demonstrated the precipitin reactions between sera from RA patients (but not controls) and EBV-infected B lymphocyte extracts [209]. That phenomenon, combined with other immunofluorescence studies, indicates the high titer of antibodies present in most of the RA patients’ sera, recognizing a nuclear antigen initially referred to as the rheumatoid arthritis nuclear antigen, which is later confirmed to be a glycine/alanine-rich repeat in EBNA-1 [209,210,211,212]. Later, Alspaugh et al. proved that the level of antibodies against EBV antigens in the synovial fluid was also higher in RA patients than in controls [213]. In addition to antibody responses, cell-mediated responses to the EBV lytic cycle antigens BZLF1 and BMLF1, which are crucial in controlling virus dissemination, may influence synovial inflammation in RA patients [214]. 

Molecular mimicry is one of the potential mechanisms explaining the correlation between EBV infection and RA onset. Sequence homology exists between the EBV-encoded protein gp110 and the QKRAA amino acid motif of HLA-DRB1*0401, which is also known as the shared epitope [215,216]. Patients with EBV infection or prior EBV infection have serum antibodies against gp110, and their T cells recognize the QKRAA motif in both HLA-DRB1*0401 and gp110 [215]. Several amino acid motifs are shared between EBNA-6 and HLA-DQ*0302, which may induce autoantibodies [217]. The glycine/alanine-rich repeat found in EBNA-1 crosslinks with cytoskeleton proteins such as type-II collagen, cytokeratin, and actin [218], and antibodies against this repeat also cross-react with a 62-kDa protein present in the synovial fluids of RA patients [219,220,221]. Antibodies against another major epitope of EBNA-1, EBV peptide p107, also recognize denatured collagen and keratin [222]. 

Furthermore, citrullinated proteins are the potential arthritogenic autoantigens in RA, and EBV infection can induce antibodies toward citrullinated proteins [223]. Antibodies against citrullinated human fibrin (AhFibA) may cross-react with a citrullinated peptide derived from EBNA-1 (EBNA35-58Cit) [224,225]. Cornillet et al. demonstrated that among anti-AhFibA-positive patients, 47% were positive for anti-EBNA35-58Cit, and nearly all (98.5%) of those positive for anti-EBNA35-58Cit were also positive for anti-AhFibA. Competition assays revealed that anti-EBNA35-58Cit antibodies exhibit significant cross-reactivity with the β60-74Cit peptide, a subset of AhFibA [224]. Cigarette smoking has the potential to trigger an HLA-DR (shared epitope)-restricted immune reaction towards citrulline-modified autoantigens, thereby contributing to the development of anti-citrulline-positive RA [226]. In addition to the above-mentioned EBV antigens, RA patients are shown to exhibit increased levels of antibodies against other EBV antigens, including EBNA-1, EBNA-2, VCA, and early antigens (EAs) [213,227,228,229,230]. 

The clonal expansion of peripheral CD8^+^CD28^−^ EBV-specific T cells, observed in patients with RA but not in controls, is believed to represent dysfunctional, senescent suppressor T cells. This phenomenon may arise from recurrent EBV stimulation and/or a primary defect in T cell differentiation and proliferation in RA [231]. Indeed, it has been demonstrated that EBV DNA loads significantly increased in peripheral blood mononuclear cells and saliva of RA patients when compared to healthy controls [232,233]. Furthermore, several studies demonstrated much higher EBV DNA and EBV mRNA levels in the synovium of RA patients compared with healthy controls [228,234,235,236]. 

In addition, impaired EBV-specific T cell function has been observed in RA patients [237,238,239]. The HLA-DRB1*04:04 shared epitope, a significant genetic predisposing factor for RA, is linked to a decreased frequency of EBV gp110 glycoprotein-specific T cells, which are essential for restraining EBV infection [214,240]. As per Scotet et al., EBV-specific T cells directed against EBV BZLF1 and BMLF1 are recruited to the synovium, potentially exacerbating the synovial inflammation characteristic of chronic RA [241]. T cell control of EBV-infected B lymphocytes is also impaired in RA patients, which leads to a rapid outgrowth of circulating EBV-infected B lymphocytes [238,242]. Those circulating B cells are more active than normal B cells, which is potentially due to similar deficiencies in the suppressor function of T cells [243]. Overall, the impairment of T cell control of B cell outgrowth correlates with the activity or severity of RA [244,245].

## 6. The Diagnosis of EBV Infection

The diagnosis of EBV infection is primarily addressed within the framework of infectious mononucleosis (IM), which is a complex and multi-factorial condition that requires a comprehensive diagnostic approach. The process typically starts with a careful consideration of the patient’s clinical history and presentation, followed by an array of laboratory tests to confirm the diagnosis.

Clinical presentation is often the first clue to an EBV infection. Adolescents and young adults who present with a sore throat, fever, malaise, lymphadenopathy, and pharyngitis are more likely candidates for IM [246,247]. Palatal petechiae, splenomegaly, and posterior cervical adenopathy are strong indicators of IM, while the absence of cervical lymphadenopathy and fatigue are less likely to be diagnosed [248,249]. Importantly, those symptoms can overlap with other viral or bacterial infections, such as streptococcal infection, so a thorough evaluation is essential.

Laboratory findings provide crucial evidence for an EBV diagnosis. Lymphocytosis, characterized by an absolute count > 4500/μL or a differential count > 50% on a peripheral smear, is a significant diagnostic marker. Moreover, the presence of atypical lymphocytes, representing more than 10% of total lymphocytes, supports the diagnosis of EBV infection. However, atypical lymphocytes are not exclusive to EBV, as they can occur in other conditions such as toxoplasmosis, rubella, and viral hepatitis [250]. Additionally, older individuals may have fewer atypical lymphocytes in their blood [251].

The heterophile antibody test, which detects antibodies that can react to antigens from unrelated species, is a valuable diagnostic tool for EBV infection [252]. Tests such as the classic Paul–Bunnell test and the “Monospot” test can confirm the presence of reactive heterophile antibodies in a patient who has compatible symptoms [252,253]. ELISA (enzyme-linked immunosorbent assay) techniques can also be used as a rapid diagnostic test. However, the “Monospot” test can be insensitive in early infection and in young children, necessitating repeat testing or EBV-specific antibody testing in certain cases [254,255].

EBV-specific antibodies are the gold standard for IM diagnosis [256]. There are two key antigens in the corresponding antibodies: the viral capsid antigen (VCA) and the Epstein–Barr nuclear antigen (EBNA). IgM-VCA antibodies, indicative of acute infection, appear at the onset of clinical illness and wane within about three months, while IgG-VCA antibodies persist for life. EBNA antibodies appear at 6 to 12 weeks after symptom onset and also persist throughout life; their presence early in the course of an illness effectively excludes acute EBV infection.

The diagnosis of EBV infection is almost certain in the presence of IgM-VCA and the absence of IgG-EBNA antibodies. In certain cases, detecting EBV DNA through polymerase chain reaction assays on blood or plasma may provide further diagnostic information [257,258]. 

Overall, diagnosing an EBV infection is a multifaceted process that involves clinical symptoms, laboratory findings, and patient history. Confirmatory tests, such as heterophile antibody tests and EBV-specific antibodies, are crucial for an accurate diagnosis. This comprehensive diagnostic approach enables healthcare providers to inform patients of their condition, associated risks, and potential treatment options.

## 7. Conclusions

EBV, one of the most prevalent human viruses worldwide, has been extensively studied, and its role in various diseases has been well-documented over the decades. It is indisputable that EBV is a significant player in infectious mononucleosis and is strongly associated with various cancers. Its involvement in autoimmune diseases like multiple sclerosis has also been a focal point of research, although the exact mechanisms remain elusive. The primary proteins involved in EBV latency and several proteins active in its lytic cycle are key contributors to EBV-related oncogenesis. As such, these proteins are targeted for the development of therapeutic vaccines aimed at enhancing T cell immunity against EBV-linked cancers. Currently, vaccination strategies against EBV are under research and have yet to achieve full success, demonstrating the challenges posed by the virus’s ability to evade the immune system and establish a lifelong latent infection.

In conclusion, while EBV is highly common and typically harmless, it poses significant health risks through its links to certain cancers and autoimmune diseases. Its insidious nature and persistent latency complicate efforts to develop effective preventive and therapeutic strategies. As we move forward, a more comprehensive understanding of EBV’s pathogenesis and immune evasion mechanisms is vital to improving outcomes for individuals affected by EBV-associated diseases.

## Data Availability

All relevant materials are available from the authors.

## Abbreviations

BCR	B cell receptor
CTAR	C-terminal activation regions
CRYAB	Alpha-crystallin B chain
CNS	Central nervous system
EBV	Epstein–Barr virus
EBNA	Epstein–Barr virus nuclear antigen
EphA2	Ephrin receptor tyrosine kinase A2
GC	Germinal center reaction
GlialCAM	Glial cell adhesion molecule
gPs	Envelope glycoproteins
gp350/220	Glycoprotein 350/220
ITAM	Immunoreceptor tyrosine-based activation motif
IL-10	Interleukin-10
IRFs	Interferon regulatory factors
IM	Infectious mononucleosis
LMP	Latent membrane protein
MBP	Myelin basic protein
MS	Multiple sclerosis
MHC I	Major histocompatibility complex class I
MHC II	Major histocompatibility complex class II
miRNAs	microRNAs
MAPK	Mitogen-activated protein kinase
NPCs	Nasopharyngeal carcinomas
NK cell	Natural killer cell
PRRs	Pattern-recognition receptors
PAMPs	Pathogen-associated molecular patterns
SLE	Systemic lupus erythematosus
TLR9	Toll-like receptor 9
TAAs	Tumor-associated antigens
VCA	Viral capsid antigen
